# *Klenkia terrae* resistant to DNA extraction in germ-free mice stools illustrates the extraction pitfall faced by metagenomics

**DOI:** 10.1038/s41598-020-66627-0

**Published:** 2020-06-23

**Authors:** Julien Andreani, Matthieu Million, Jean-Pierre Baudoin, Yusuke Ominami, Jacques Yaacoub Bou Khalil, Cécile Frémond, Saber Khelaifia, Anthony Levasseur, Didier Raoult, Bernard La Scola

**Affiliations:** 1 Aix Marseille Univ, IRD, APHM, MEPHI, Marseille, France, Marseille, France; 2 Hitachi High-Technologies Corporation, Science & Medical Systems Business Group, 24-14, Nishi-shimbashi 1-chome, Minato-ku, Tokyo 105-8717 Japan; 3 Institut Hospitalo-Universitaire (IHU) - Méditerranée Infection, Marseille, France; 4 Phenomin-TAAM, UPS44, Centre National de la Recherche Scientifique, Orléans, France

**Keywords:** Metagenomics, Bacteriology

## Abstract

Over the past decade, metagenomics has become the preferred method for exploring complex microbiota such as human gut microbiota. However, several bias affecting the results of microbiota composition, such as those due to DNA extraction, have been reported. These bias have been confirmed with the development of culturomics technique. In the present study, we report the contamination of a gnotobiotic mice unit with a bacterium first detected by gram staining. Scanning electron microscopy and transmission electron microscopy permitted to detect a bacterium with a thick cell wall. However, in parallel, the first attempt to identify and culture this bacterium by gene amplification and metagenomics of universal 16S rRNA failed. Finally, the isolation in culture of a fastidious bacterium not detected by using universal PCR was successfully achieved by using a BCYE agar plate with CO_2_ atmosphere at 30 °C. We performed genome sequencing of this bacterium using a strong extraction procedure. The genomic comparison allowed us to classify this bacterium as *Klenkia terrae*. And finally, it was also detected in the stool and kibble that caused the contamination by using specific qPCR against this bacterium. The elucidation of this contamination provides additional evidence that DNA extraction could be a bias for the study of the microbiota. Currently, most studies that strive to analyze and compare the gut microbiota are based on metagenomics. In a gnotobiotic mice unit contaminated with the fastidious Actinobacteria *Klenkia terrae*, standard culture, 16S rRNA gene amplification and metagenomics failed to identify the micro-organism observed in stools by gram-staining. Only a procedure based on culturomics allowed us to identify this bacterium and to elucidate the mode of contamination of the gnotobiotic mice unit through diet.

## Introduction

In an attempt to explore bacterial, environmental or vertebrate-associated flora, now called microbiota, microbial culture was the first and for a long time the only technique to be used^1,2^. This period was characterized by use of complex, expensive, often highly smart, and innovative but also highly time consuming approaches. This approach has been strongly modified when molecular methods arrived, especially 16S rRNA gene amplification, cloning and sequencing at the end on the twentieth century^3^. Metagenomics has then become a useful method for exploring complex ecosystems, from the environment to the human microbiota^4^. From these first studies, it became clear that a large part of the microbiota, whether environmental or associated with vertebrates, was totally unknown. This “dark matter” has been described as uncultivable^5,6^. In recent years, the opinions of the scientific community have gradually evolved. Indeed, it has been shown that metagenomics is associated with many biases such as the choice of primers to amplify 16S rRNA sequences or low depth of sequencing^5^. These biases have been partially overcome by replacing 16S rRNA-based sequencing with high throughput sequencing of total DNA extracts and by increasing the depth of sequencing allowed by the latest generation sequencers^5^. However, a bias that not disappeared is that of DNA extraction associate with the process of amplification and sequencing^5^. This has become evident with the development of culturomics, a technique multiplying culture approaches to explore the bacteria repertoire on a given microbiota such as the human gut^5,7,8^. In the first work establishing the name culturomics^8^, later confirmed in dozens of studies, it became evident that despite the considerable progress in metagenomics that has made it possible to extend the definition of the various microbiota repertoires, a large part of the cultivated repertoire has been forgotten by metagenomics^5^.

TAAM laboratory (http://transgenose.cnrs-orleans.fr/eng/taam/presentation.php) has a germ-free service unit and realises stool control by gram staining. During routine survey of germ-free mice, probable bacterial contaminants have been observed in stools using gram staining. Standard culture on routine media and universal 16S rRNA amplification failed to detect directly any bacterial contaminant in the stool samples. The possibility of contamination by a non-bacterial micro-organism such as a giant virus has been suspected^9^. Indeed, this first giant virus isolated was long considered a gram positive intra-amoebal bacterium resistant to 16S rRNA amplification^10^. As a laboratory specializing in giant viruses and the culture of tedious bacteria, we received up to 30 samples of gnotobiotic mouse stools and their kibbles to determine the nature of the contaminant. These samples were investigated through different approaches such as, culturomics^7^, co-culture on amoeba^9^, metagenomic analyses and electronic microscopy (transmission TEM and by scanning SEM). The isolation of a gram-positive bacterium resistant to DNA extraction has confirmed once again that extraction bias remains a major limitation to microbiota exploration that can in part be circumvented by the culturomics approach.

## Results

### Stools

We summarize all samples tested in this study in Table 1.

**Table 1:** Samples investigated by multiple approaches.

Samples and their names	Number of sample	Method used
26.2.	1	Scanning Electron microscopy (SEM) qPCR*
Iax_3, 26.1	2	Metagenomic qPCR*
17.9, 26.3	2	Embedding (TEM) Culture
Iax_1, Iax_2, Iax_4, 1, 2, 3, 4, 5, 6, 7, 8, 9, 10, 11, 12, 13, 14, 15, 16, 17, 18, 19, 20, 17.1, 17.2, 17.4, 26.4, 26.5	28	qPCR*
10 kibbles	10	Culture

In the table “*” was employed for qPCR designed after the genome sequencing and targeting the bacterium isolate.

### Detection of micro-organisms by scanning electron microscopy (SEM) and transmission electronic microscopy

**Fig. 1:**
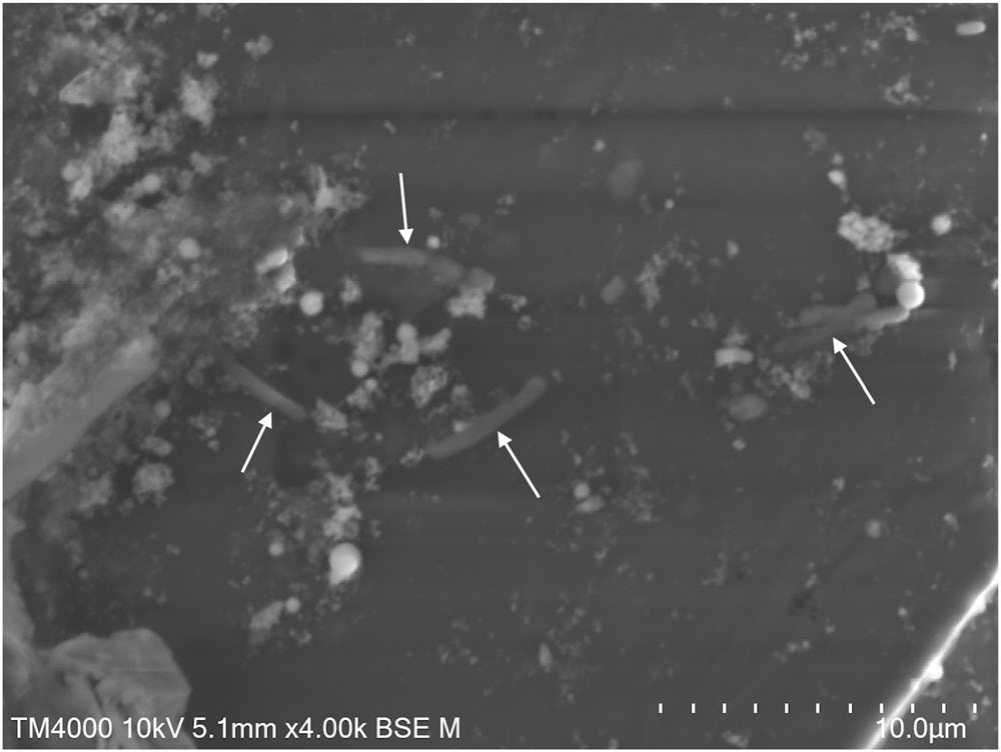
Electron SEM radiographs of bacteria present in sample 26.2 of germ-free mice stool. Technical settings and scale bars are indicated in the images. White arrows indicate micro-organisms.

**Fig. 2:**
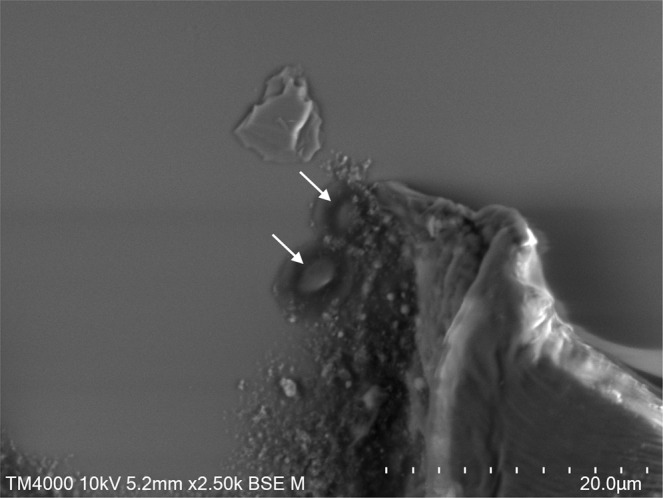
Electron SEM radiographs of bacteria present in sample 26.2 of gnotobiotic mice stool. Technical settings and scale bars are indicated in the images. White arrows indicate micro-organisms.

Bacteria were detected in the stool sample 26.2 of germ-free mice by SEM strategy. We noticed the presence of elongated-bacilli shaped microorganisms with variable dimensions ranging from 1μm to 3μm in height and with a diameter of about 500 nm (Fig. 1). Some particles appeared more ovoid with a size of about 3 μm by 1,5 μm in diameter (Fig. 2).

**Fig. 3:**
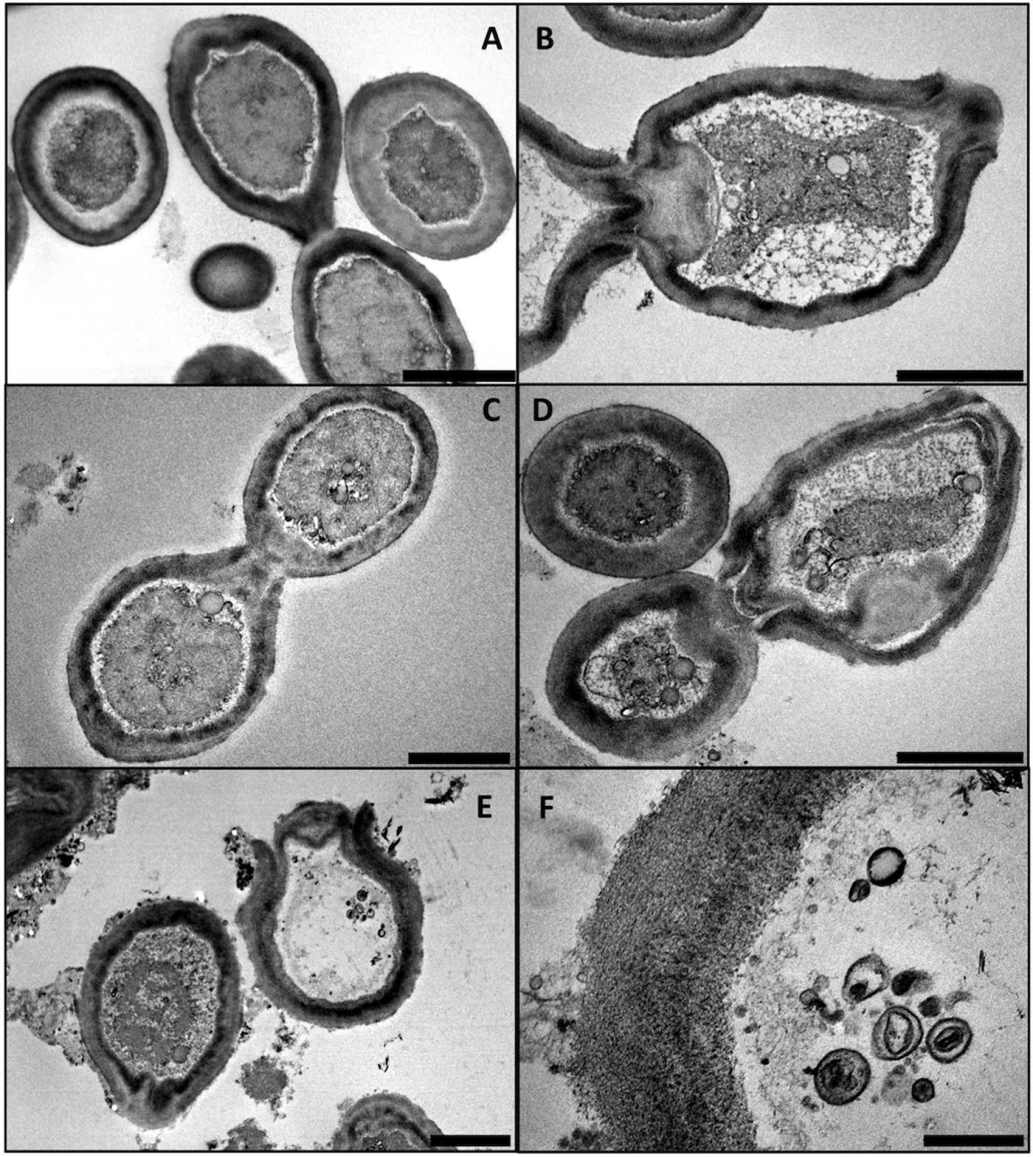
Micrographs obtained by Transmission electron microscopy in the stool embedding 26.3 and 17.9. Scale bars represent 2μm except for the panel (F) scale bar indicates 500 nm. (**A**–**D**) were observed for the 17.9 stool sample. (**E**) and (**F**) were observed for the 26.3 sample, and (**F**) was a higher magnification of (**E**). All images were captured on Morgagni device.

The stools 26.3 and 17.9 were examined under transmission electron microscopy and allowed the observation of similar multiple budding rod bacterial bodies (Fig. 3). The cell wall appears complex, unusually large but with variable width. From the inside to the outside of the bacteria cells, we observed multiple successions of dense and hyper-dense layers. We observed a large layer measuring in total a range from 400 to 650 nm. Bacterial particles present a size ranging from 1 to 3 μm.

### Metagenomic analyses performed on the stools 26.1 and IAx3

In the meantime, two stools, from 2 different isolators, IAX_3 (named S1) and 26.1 (named S2), were used for metagenomic analyses. We obtained, for S1, 104 contigs representing 168,142 base pairs with a N50 of 1,528 bp and for the S2 sample, 134 contigs representing 228,556 bp with a N50 of 1,578. We sorted all the contigs on average coverage parameters filtering and conserving contigs only when contigs have an average coverage greater than or equal to 5,0. Finally, 74 contigs were conserved for the stool IAX-3 and 90 contigs for the sample 26.1. Some of them presented a best hit result with the host *Mus musculus* (Supplementary file S1) (5 and 4 contigs respectively). For the sample IAX3, the majority of contigs (43 on 74) were identified as *Escherichia coli*, some other have a best hit with *Ralstonia* sp. and *Ralstonia* phage. Concerning the sample 26.1, cloning vector is a major contaminant (with 1 contig and 23,000 reads). Some contigs are identified as *Ralstonia* sp. and *Ralstonia* phage and as *Methylobacterium* spp. and *Escherichia coli* were also found.

Total read numbers was dominated by host reads (*Mus musculus*) and by *E. coli* reads. Indeed, we had 31,966 reads on the 58,433 for IAx_3 and 16,551 on the 18,281 for the sample 26.1 attributed to *M. musculus* and *E. coli* when we excluded the cloning vector contig.

### Culture isolations and bacterial assays

All culture attempts were negative except culture on BCYE at 30 °C. Under these conditions, after 14 days, we observed growth of white colonies on the BCYE agar plates, from 2 different isolators numbered 17.9 and 26.3. No growth was observed on the 35 °C plates. A few days after, the colonies took on a pink pigmentation. Inoculation of the industrial food (kibble) given to germ-free mice under the same conditions allowed the isolation of the same typical bacteria. A partial 16S sequencing (879 nucleotides) performed on the pink colonies has a best hit in blast nucleotide on 877 base pairs with 99% of identity with *Geodermatophilus* sp. strain YIM_M1315 (accession number: LT608342.1) and another strain with the accession number LT746188.1 proposed as *Klenkia marina* sp. nov.^11^. In the meantime, we have performed some sensitivity tests on our isolate by autoclave testing and have not found any particular resistance under the conditions tested.

### Genome analy**s**is

**Fig. 4:**
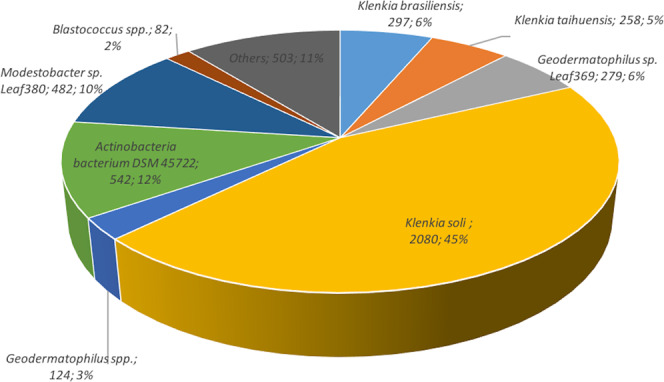
Representation of the best hit obtained by blastp based on the predicted Klenkia terrae IHUMI-26.3 proteins. 4,647 proteins were blast against NCBI database. % and values of hits’ number are indicated for each labels.

**Fig. 5:**
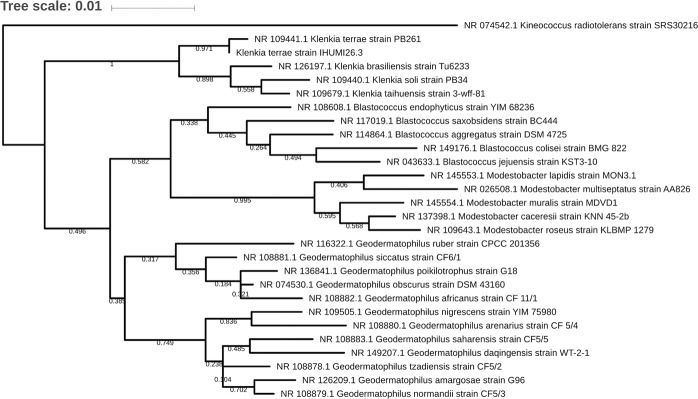
Phylogenetic tree based on the 16S rRNA gene. Maximum likelihood tree was built using MEGA6.0 package program with Jukes cantor method and 1,000 bootstrap replicates. The alignment was performed using MUSCLE and visualisation was done using iTol program.

**Fig. 6:**
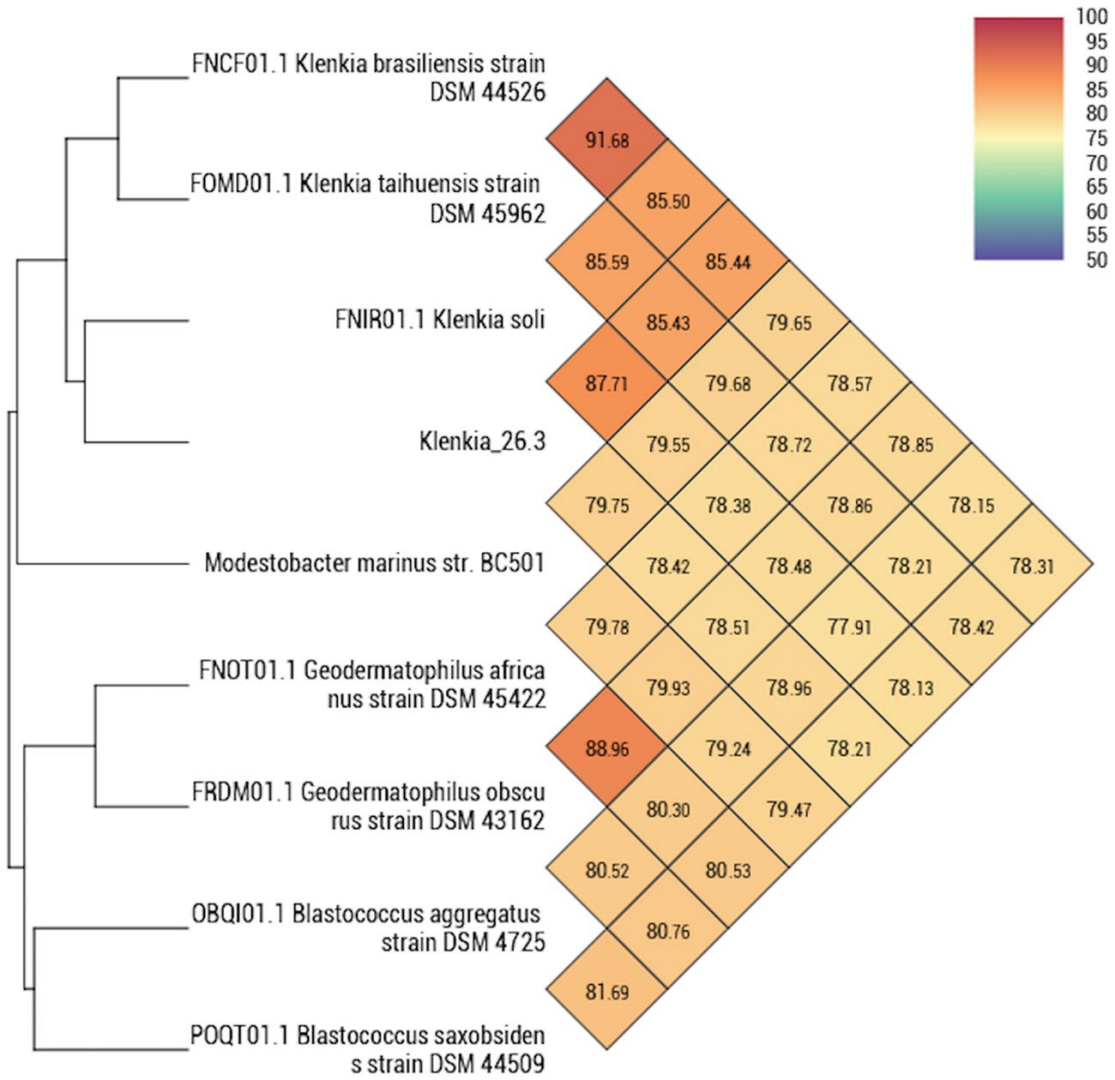
Heatmap obtained for Klenkia spp. and other associated bacteria. Values indicate the percentage of identity between the different strains.

We obtained a draft genome of 23 scaffolds representing 4,806,923 base pairs with a major one of 4,731,847 base pairs. Prodigal predicted 4,679 proteins, 4,594 of them being including into a major scaffold plus 48 tRNAs and 4,647 of them having a result in the nr database. The large majority of the best matches were obtained with *Klenkia soli* (2080 hits), and 958 with various *Geodermatophilus* spp. (Fig. 4). Regarding best hits’ distribution, we observed that *Klenkia* 26.3 genome isolated from germ-free mice is a close genome of another strain named *Klenkia soli*. 26.3 bacterium deposited in CSUR (http://www.mediterranee-infection.com/article.php?laref=14&titre=collection-de-souches) culture collection under number CSURP6454. However, the complete 16S rRNA gene extracted from the complete genome of Klenkia 26.3 is close to *Klenkia terrae*, for which no genome is currently available (Fig. 5). We decided to name this bacteria *Klenkia terrae* strain IHUMI-26.3. Using Mauve aligner program, we visualised a close proximity, even if some blocks seem absent in *Klenkia* 26.3 genome (data not shown). We determined 156,796 single-nucleotide polymorphisms between different blocks aligned between *K. soli* DSM 45843genome and *K. terrae* IHUMI-26.3 genome. Analysis by OrthoANI confirmed the close relationship between genomes of *K. soli* strain DSM 45843 and *K. terrae* IHUMI-26.3 (Fig. 6).

### qPCR on stool samples retrospectively used on stool sample

After the failed detection of the bacterium using 16S rRNA gene amplifications and the isolation of the bacterium. We designed 2 different specific primers against Rpob and gyrase subunit A genes of *Klenkia* spp. and tested samples S1 and S2 used early for metagenomic. All qPCR are positive for the sample S2 (sample 26.1) where both primers and sequencing confirmed the presence of *K. terrae* IHUMI-26.3. qPCR with the same primer was tested against more stool samples from other isolators. Nine stools were positive in qPCR (data not shown), thus revealing a large contamination of the mice unit by this strain due to kibbles consumption. It also revealed that the universal 16S rRNA can’t detect the bacterial presence directly in feces samples.

### Metagenomic analyses on artificial bacterial mixture

To explain the non-detection of *Klenkia* sequences by metagenomic performed directly on the stool samples, we suspected a DNA extraction problem.

An artificial bacterial mix was used to investigate this matter. We observed that the number of scaffolds obtained is 20 times higher in artificial mix compared to the metagenomic shotgun made on the 2 stools even if we have the same theoretically DNA quantity used as input (1 ng). In artificial bacterial mixtures A and B (supplementary file S2), we sorted the contigs on average greater than 5 (as we did previously for stool samples). We observed a large difference between mixture A and B in the number of read despite the fact that we had about the same number of scaffold. For mixture A, one third (≈36,4%) of the scaffold’s number were identified as *Klenkia* spp. Sequences and represented only ≈13,1% of the total reads number. For mixture B, nearly half (≈46,3%) of the scaffold’s number were identified as *Klenkia* spp. sequences and represent only ≈5,8% of the total number of reads. Altogether, these elements and the difference observed between stool and artificial metagenomic, highlight a deep bias in the sequencing and in the read coverage. This difference could explain why in this case, *Klenkia* spp. reads were omitted in the stool in the absence of deep metagenomic sequencing.

## Discussion

In this work, we were unable to detect *Klenkia* spp. in our metagenomic analyses and by pan-16S rRNA PCR, but we succeeded in isolating the bacterium that contaminated the mice unit using BCYE agar plate, low temperature and a long-time incubation. It was identified as *Klenkia terrae* using the current standard by 16S comparison obtained after the whole-genome sequencing. The universal 16S PCR probably failed to amplify Klenkia DNA because we failed to extract DNA in mice stools. Indeed, Klenkia 16 s rRNA sequences matched perfectely with 536F and rp2 primers (Supplementary file S3). A recent study, comparing 10 different extraction methods performed on the same stool samples, showed that the results were never identical. In addition, mechanical lysis of stools followed by a glycan degradation step was more efficient than any other extraction method at obtaining an optimized liberation of DNA from stool exopolysaccharides^12^. In addition, we were aware of the difficulty of cross-referencing data from culturomics and metagnomics studies^8^. Nevertheless, specific primers and PCR sequencing allowed us to detect the DNA of *Klenkia* in the same DNA that we used for metagenomic sequencing and analyses. First of all, we were surprised by the small quantities of reads obtained. Finally, we can’t clearly interpret this. It could be a mix between bias of DNA extraction, due to the particular thick membranes of *K. terrae* IHUMI-26.3, and bias in depth coverage during the whole genome shotgun (WGS) sequencing that led to the recovery of reads corresponding to mice and contaminant DNA. This was likely worsened by the high GC% content of *K. terrae* at 75%. Indeed, high GC content organisms are reported to be more difficult to sequence compared to organisms possessing a moderate GC content^13,14^.

*K. terrae* is a fastidious gram-positive bacterium belonging to the Actinobacteria phylum. The majority of *Geodermatophilus* spp. and *Klenkia* spp. isolates came from arid soils of multiple extreme environments^15^, but also from grass or “classic” soils^16,17^. On the other hand, the close-genera *Blastoccocus* and *Modestobacter* have been isolated to date from stones^18^. Through genome sequencing, we have unambiguously identified our isolate as a member of the *Geodermatophilaceae* according to the recent taxonomic suggestion of Montero-Calasanz et *al*. in the Geodermatophiliales order^11^. Concerning bacterial morphologies, an ultrastructure study conducted in 1970^19^ described that the strain *Geodermatophilus* sp. 22-68, isolated form the Mount Everest soil took in high-altitude, presented two forms. Indeed, the first one, C-form, is a coccoid one and the second, R-form, is a motile budding rod one. It is interesting to note that it was the use of the media that made it possible to adjust the R or C forms obtained. In our stools observations, we only observed R-forms, even if the fibrous layer could be visualised. It is striking to note that the size of the layers can increase from 10 nm to nearly 600 nm in our observations. This phenomenon needs to be further investigated with *Geodermatophilus* and *Klenkia* bacterial descriptions.

Finally, Klenkia DNA has also been detected by specific designed qPCR in nine other stool samples and revealed a large contamination in the livestock. The primers of PCR system allowed us to increase the sensitivity compared to the single metagenomic analysis, where we identified numerous contigs as the host (*Mus musculus*) and as *Escherichia coli*. We identified the diet as the source of contamination and tried to understand this. Among the *Geodermatophiliales* order, inconstant resistances between species were observed, concerning notably the tolerance at radiation level, heavy metals and reactive oxygen species^15^. Thermal resistance has not been investigated in this group of bacteria. We performed some tests in autoclave but we could not demonstrate the resistance of *Klenkia terrae* to this test. As data of the sterilization process of kibbles could not be retrieved, we could not exclude that the sterilization procedure failed upstream. Such contamination of animals has already been observed in germ-free mice units^20^. The authors pointed out that observation of bacteria in stools was not synonymous with bacterial colonization, e.g the ingestion of dead bacteria through the diet could be observed in the stool thanks to gram staining. However, in 1985, a mono-contamination with a filamentous bacillus was observed by gram staining in stools of germ-free mice and the anaerobic bacterium was further isolated in thioglycollate broth, but could not be identified^21^. Finally, the authors concluded that this contamination, as in our case, is due to animal feed, even if the sterilization was carried out by an industrial company. The use of Scanning Electron Microscopy using the Hitachi TM4000 tabletop microscope with a reduced vacuum level (100 Pa to 101 Pa) obtained after only 2 minutes and requiring no sample preparation seems to be appropriate to easily and quickly detect and confirm the presence of bacteria. This technique could be associated with gram staining in variety of applications, such as clinical or microbiological research, especially when a rapid diagnosis and detection are required. In germ-free laboratory, daily controls of mice stools combining gram staining, table-top microscope and universal 16S rRNA amplification might be an additional and efficient solutions to manage quality.

In our case, metagenomic detected few bacterial DNA read, in that case probably in transit from diet, but did not detect the fraction corresponding to the unique live bacterium.

## Materials and methods

### Stool samples and various strategies

TAAM laboratory sent to the IHU Méditerrannée-infection 31 samples of mice stool from 4 different distant isolators and 10 kibbles. TAAM is a laboratory doing breeding mice with agreement E-45-234-006 provided by the French ministry of agriculture in accordance to national relevant guidelines and regulation. In the present case, the group of mice that had their stool analyzed were not part of an experimental protocol. The autoclaved kibbles came from preserved foods given to mice weeks earlier. All stool samples were re-suspended in phosphate buffered saline and sterile beads were used to homogize samples. For the 10 kibbles, they were homogenized into one tube with 20 mL of Trypticase soja browth (Oxoïd^TM^).

### Electron microscopy (SEM and TEM)

Samples 26.1 and 26.2 were directly suspended in 2.5% of glutaraldehyde fixative solution, and then we directly smeared samples onto microscopy slides and proceeded to images acquisition without any additional staining on a tabletop scanning electron microscope SEM (Hitachi TM4000) with approximately 60 centimeters in height and 33 cm in width to evaluate bacterial structures.

For the stool embedding for TEM analysis, two samples 17.9 and 26.3 were fixed overnight in 2.5% glutataldehyde in 0.1M cacodylate buffer at 4 °C. Then, the samples were washed three times for 10 minutes with 0.2 M saccharose in 0.1M cacodylate buffer. They were post-fixed for 1 hour at room temperature with 1%OsO4 in 1.25% Potassium Ferrocyanate/0.1M cacodylate solution and were then washed three times for 10 minutes with distilled water, and gradually dehydrated with increasing concentrations of ethanol in water: 25%, 50%, 75%, 90%, 99% and 100% ethanol during 10 min, 3 min, 3 min, 10 min, 10 min, and 30 min, respectively. Resin substitution was achieved by incubating the samples in successive 15 minutes baths in mixtures of Epon812 resin and 100% ethanol solution, with respective proportions of 25%/75%, 50%/50%, 75%/25%, and overnight in 100% Epon812 resin. Finally, samples were placed in 350 µL of 100% fresh Epon812 resin and polymerization was achieved at 60 °C for 3 days. Between all steps, the samples mentioned above were centrifuged at 5 000 g and supernatants were discarded. Ultrathin sections (70 nm) were cut on a UC7 (Leica) ultramicrotome and deposited on 300 mesh copper/rhodium grids (Maxtaform HR25, TAAB). Sections were post-stained with 5% uranyl acetate and lead citrate according to the Reynolds method^22^.

Electron micrographs were obtained on a Morgani 268D transmission electron microscope operated at 80 keV. ImageJ (https://imagej.nih.gov/ij/) software was used to determine particle size in embedding and in negative staining.

### Culturomics, amoeba co-culture, first PCR detection and autoclave susceptibility testing

Following an earlier expertise of the culturomics procedure on stool samples^7,8^, aerobic and anaerobic conditions were used and incubated at 37 °C. Firstly, PCR against 16S rRNA gene were done using following primers 536F-CAGCAGCCGCGGTAATAC and rp2-ACGGCTACCTTGTTACGACTT using the same protocol of Morel *et al*.^23^. Briefly, no 16S sequences were obtained in mice stools by this method. In parallel, based on a previous experience with fastidious bacteria^24^, we added supplementary media and conditions: BCYE agar plate (Oxoïd; UK) and COS agar plate (Oxoïd; UK) at 35 °C and 30 °C with CO_2_ generator (ThermoFisher, MA, USA).

Amoeba co-culture was attempted to isolate potential giant virus or intracellular bacterium growing in amoeba^9,25,26^. We used two amoebas as cell support: *Acanthamoeba castellanii* strain Neff (ATCC 30010) and *Vermamoeba vermiformis* strain CDC19. We followed the classic steps of our co-culture procedure, but we avoided using antibiotics^27^.

For autoclave essays, suspension of the isolated bacterium was performed in 1 mL of trypticase soja browth (Oxoïd; UK). We used various conditions of temperature independently with 70, 80, 90, 100, 110 and 121 °C during 15 minutes. After each cycle, the 1 mL was inoculated on the BCYE agar plate under CO_2_ atmosphere at 30 °C.

### Metagenomic sequencing and assembly of 2 stool samples

Two stools from 2 different isolators, T3AX_5 and 26AX_26.1, were first extracted by a mechanical treatment performed by powder glass beads acid washed (G4649–500g Sigma) and 0,5 mm glass beads Cell disruption media (Scientific Industries, Inc) using a FastPrep BIO 101 instrument (Qbiogene, Strasbourg, France) at maximum speed (6.5 m/sec) for 90 seconds. Then, the stools were treated with two types of lyses: NucleoSpin Tissue kit (Macherey Nagel, Hoerdt, France) and deglycosylation step followed by the EZ1 Advanced XL device (Qiagen, Courtaboeuf, France)^12^.

Two paired end libraries (samples T3AX_5 and T26AX_26,1) were constructed according to the Nextera XT protocol (Illumina). The tagmentation step fragmented and tagged the DNA. Then, limited-cycle PCR amplification (12 cycles) completed the tag adapters and introduced dual-index barcodes. After purification on AMPure XP beads (Beckman Coulter Inc, Fullerton, CA, USA), the libraries were normalized on specific beads and were then pooled for sequencing on the MiSeq. Automated cluster generation and paired end sequencing with dual index reads were performed in a 2 × 250-bp run, with a cluster passing quality control filters of 94.9%. The assembly was performed on CLC genomics workbench 7.5 (https://www.qiagenbioinformatics.com/products/clc-genomics-workbench/) with standard parameter, with a bubble size of 50 and a word size maximal at 64 and with a minimum length of contigs of 1,000 base pairs.

### Metagenomic sequencing on artificial mixture of bacteria

Strains of *Staphylococcus aureus* (CIP7625) *Escherichia coli* (CIP7624) and *Clostridium butyricum* NEC8 (accession number: GCA_001458815.1) were used in association with the bacterium isolate 26.3. After 7 days, when colonies were visible for the four bacteria, we picked one colony per each 4 bacteria and suspended it in 1 ml of PBS buffer to make an artificial mix and named it mix A. The same procedure of picking was reproduced 2 weeks later to simulate artificial aging of bacteria and was named mix B.

Genomic DNA was sequenced on the MiSeq Technology (Illumina Inc, San Diego, CA, USA) with the paired-end application as described above. Briefly, DNA of mix A and B were quantified by a Qubit assay with the high sensitivity kit (Life technologies, Carlsbad, CA, USA) to 3.3 and 2.9 ng/µl respectively. Total information of 6.8 Gb was obtained with a cluster density of 1,167,000 per mm^2^ with a cluster passing quality control filters of 61.2% (13,229,000 passed filtered clusters). Within this run, the index representation for the mix A and B was established at 2.05 and 7.77%. Therefore, respectively 271,790 and 1,027,641 paired-end reads were trimmed and filtered according to the read qualities.

The assembly was done on CLC using standard parameter (as see in previously section).

### Sequencing, genome assembly and annotation of the bacterium isolate

Genome DNA (gDNA) of the isolated bacterium strain IHUMI-26.3 was extracted by a mechanical treatment performed by powder glass beads acid washed (G4649–500g Sigma) using a FastPrep BIO 101 instrument (Qbiogene, Strasbourg, France) at maximum speed (6.5) for 90 seconds. After a 2-hour lysozyme incubation at 37 °C, DNA was extracted on the EZ1 biorobot (Qiagen) with the EZ1 DNA tissues kit. The elution volume was 50 µl. gDNA was quantified by a Qubit assay with the high sensitivity kit (Life technologies, Carlsbad, CA, USA) to 71.5 ng/µl

Genomic DNA was sequenced using the MiSeq (Illumina Inc, San Diego, CA, USA) with the same protocol that we previously used^28^. As for the genomic assembly, we used paired-end reads (2,454,522 reads) after trimming the reads on the program CLCgenomics workbench 7.5. The assembly was performed on CLC using standard parameter (a bubble size of 50 and a word size maximal at 64 and with a minimum length of contigs of 1,000 base pairs).

Genes prediction was performed using Prodigal^29^. Blastp against non-redundant database was done with an e-value cut-off at 10^−2^. tRNAs were predicted using bacteria tRNA-scan online parameter^30^. Annotation was performed based on the blastp results and Interproscan v66.0 https://www.ebi.ac.uk/interpro/search/sequence-search. The 23 scaffolds of the genome were available under the accession number: UEXK01000001-UEXK01000023 on the embl/EBI database. Mauve program was used to align genomes^31^ and OrthoANI to perform genomic comparison^32^.

### Alignment of 16S rRNA sequences

The 16S rRNA sequence obtained from the culture of Klenkia sp. was aligned with the primers cited on a previous section (536F and rp2) and with the sequence of our *Klenkia terrae* strain. For that, we used Muscle on MEGA 6.06^33^. We used this latter alignment on MVIEW^34^ online https://www.ebi.ac.uk/Tools/msa/mview/.

### Detection of Klenkia sp. by qPCR

Specific primers were designed against Rpob and Gyrase subunit A genes based on *Klenkia* spp available in NCBI database and with our *Klenkia* genome sequencing using Primer-blast^35^. DNA extraction was performed using EZ1 DNA tissue kit (Qiagen) with Bacterial card and EZ1 automate.

For the qPCR detection, we used the same primers. The DNA was amplified with the LightCycler 480 SYBR green I Master (Roche). The reaction mixture (20 µL) per sample was prepared as follows: DNA template (5 µL), forward primer (1 µL, 10 µM), reverse primer (1 µL, 10 µM), master mix (2×, 10 µL) and DEPC-treated water (3 µL). The amplification program includes an initial step of denaturation at 95 °C for 5 minutes followed by 45 cycles; each cycle consisted of denaturation at 95 °C for 10 seconds, annealing at 60 °C for 20 seconds and extension at 72 °C for 30 seconds, then single cycle of melting curve step followed by cooling.

The real time amplification was performed using CFX96 real time (Bio-Rad®, France).

The results of real time amplification were analysed by Bio-Rad CFX Manager (Bio-Rad).

## Data availability

All data and material available

## Supplementary information

Supplementary file S1.

Supplementary file S2.

Supplementary file S3.

## References

[1] Savage DC, Dubos R, Schaedler RW (1968). The gastrointestinal epithelium and its autochthonous bacterial flora. J. Exp. Med..

[2] Finegold SM, Attebery HR, Sutter VL (1974). Effect of diet on human fecal flora: comparison of Japanese and American diets. Am. J. Clin. Nutr..

[3] Eckburg PB (2005). Diversity of the human intestinal microbial flora. Science.

[4] Turnbaugh PJ (2010). Organismal, genetic, and transcriptional variation in the deeply sequenced gut microbiomes of identical twins. Proc. Natl. Acad. Sci. USA.

[5] Lagier J-C (2018). Culturing the human microbiota and culturomics. Nat. Rev. Microbiol..

[6] Rinke C (2013). Insights into the phylogeny and coding potential of microbial dark matter. Nature.

[7] Lagier J-C (2016). Culture of previously uncultured members of the human gut microbiota by culturomics. Nature Microbiology.

[8] Lagier JC (2012). Microbial culturomics: paradigm shift in the human gut microbiome study. Clinical Microbiology and Infection.

[9] Khalil JYB, Andreani J, La Scola B (2016). Updating strategies for isolating and discovering giant viruses. Current Opinion in Microbiology.

[10] Raoult D, La Scola B, Birtles R (2007). The discovery and characterization of Mimivirus, the largest known virus and putative pneumonia agent. Clin. Infect. Dis..

[11] Montero-Calasanz MDC (2017). Genome-Scale Data Call for a Taxonomic Rearrangement of Geodermatophilaceae. Front Microbiol.

[12] Angelakis E (2016). Glycans affect DNA extraction and induce substantial differences in gut metagenomic studies. Sci. Rep..

[13] Shin SC (2013). Advantages of Single-Molecule Real-Time Sequencing in High-GC Content Genomes. PLoS ONE.

[14] Scott D, Ely B (2015). Comparison of genome sequencing technology and assembly methods for the analysis of a GC-rich bacterial genome. Curr. Microbiol..

[15] Gtari M (2012). Contrasted resistance of stone-dwelling Geodermatophilaceae species to stresses known to give rise to reactive oxygen species. FEMS Microbiol. Ecol..

[16] Ivanova N (2010). Complete genome sequence of Geodermatophilus obscurus type strain (G-20T). Standards in Genomic Sciences.

[17] Jin L, Lee H-G, Kim H-S, Ahn C-Y, Oh H-M (2013). Geodermatophilus soli sp. nov. and Geodermatophilus terrae sp. nov., two actinobacteria isolated from grass soil. International Journal of Systematic and Evolutionary Microbiology.

[18] Sghaier H (2016). Stone-dwelling actinobacteria Blastococcus saxobsidens, Modestobacter marinus and Geodermatophilus obscurus proteogenomes. ISME J.

[19] Ishiguro EE, Wolfe RS (1970). Control of morphogenesis in Geodermatophilus: ultrastructural studies. J. Bacteriol..

[20] Taylor DM, Read L, Neal DL (1986). Determining the viability of faecal bacteria present in germ-free mice. Lab. Anim..

[21] Taylor DM (1985). Monocontamination of germ-free mice by a fastidious unidentified anaerobe. Lab. Anim..

[22] Reynolds ES (1963). The Use Of Lead Citrate At High Ph As An Electron-Opaque Stain In Electron Microscopy. The Journal of Cell Biology.

[23] Morel A-S (2015). Complementarity between targeted real-time specific PCR and conventional broad-range 16S rDNA PCR in the syndrome-driven diagnosis of infectious diseases. Eur. J. Clin. Microbiol. Infect. Dis..

[24] Imbert G, Seccia Y, La Scola B (2005). Methylobacterium sp. bacteraemia due to a contaminated endoscope. J. Hosp. Infect..

[25] Birtles RJ (2000). ‘Candidatus Odyssella thessalonicensis’ gen. nov., sp. nov., an obligate intracellular parasite of Acanthamoeba species. International Journal of Systematic and Evolutionary Microbiology.

[26] Scola BL (2004). Legionella drancourtii sp. nov., a strictly intracellular amoebal pathogen. International Journal of Systematic and Evolutionary Microbiology.

[27] Bou Khalil, J. Y., Andreani, J., Raoult, D. & La Scola, B. A Rapid Strategy for the Isolation of New Faustoviruses from Environmental Samples Using Vermamoeba vermiformis. *J Vis Exp* e54104–e54104, 10.3791/54104 (2016). 10.3791/54104 PMC4927758 27341059

[28] Andreani J (2018). Orpheovirus IHUMI-LCC2: A New Virus among the Giant Viruses. Front Microbiol.

[29] Hyatt D (2010). Prodigal: prokaryotic gene recognition and translation initiation site identification. BMC Bioinformatics.

[30] Lowe TM, Chan PP (2016). tRNAscan-SE On-line: integrating search and context for analysis of transfer RNA genes. Nucl. Acids Res..

[31] Darling ACE, Mau B, Blattner FR, Perna NT (2004). Mauve: multiple alignment of conserved genomic sequence with rearrangements. Genome Res..

[32] Ouk Kim Y, Chun J, Lee I, Park S-C (2016). OrthoANI: An improved algorithm and software for calculating average nucleotide identity. International Journal of Systematic and Evolutionary Microbiology.

[33] Tamura K, Stecher G, Peterson D, Filipski A, Kumar S (2013). MEGA6: Molecular Evolutionary Genetics Analysis version 6.0. Mol. Biol. Evol..

[34] Brown NP, Leroy C, Sander C (1998). MView: a web-compatible database search or multiple alignment viewer. Bioinformatics.

[35] Ye J (2012). Primer-BLAST: A tool to design target-specific primers for polymerase chain reaction. BMC Bioinformatics.

