# Supplementary material for: Klenkia terrae resistant to DNA extraction in germ-free mice stools illustrates the extraction pitfall faced by metagenomics
Source: Sci Rep. 2020 Jun 23;10:10228. doi: 10.1038/s41598-020-66627-0 (PMC7311423; doi:10.1038/s41598-020-66627-0)
Supplement: Supplementary file 3 — Supplementary file S3. [file 41598_2020_66627_MOESM3_ESM.pdf]

Research article: *Klenkia terrae* resistant to DNA extraction in germ-free mice stools illustrates the extraction pitfall faced by metagenomics.

Julien Andreani[1], Matthieu Million[1], Jean-Pierre Baudoin[1], Yusuke Ominami [2], Jacques Yaacoub Bou Khalil[3], Cécile Frémond[4], Saber Khelaifa[1], Anthony Levasseur[1], Didier Raoult[1,3], Bernard La Scola[1,3#]

[1] Microbes, Evolution, Phylogeny and Infection (MEΦI), Aix-Marseille Université UM63, Institut de Recherche pour le Développement IRD 198, Assistance Publique – Hôpitaux de Marseille (AP-HM), Marseille, France.

[2] Hitachi High-Technologies Corporation, Science & Medical Systems Business Group 24-14, Nishi-shimbashi 1-chome, Minato-ku, Tokyo 105-8717 Japan.

[3] Institut Hospitalo-Universitaire (IHU) - Méditerranée Infection, Marseille, France

[4] Phenomin-TAAM, UPS44, Centre National de la Recherche Scientifique, Orléans, France

#Corresponding author: Bernard La Scola

```
Reference sequence (1): 536F-Rp2
Identities normalised by aligned length.
Colored by: identity

                                        cov    pid    1 [        .         .         .         .         :         .         .         . 80
1 536F-Rp2                           100.0% 100.0%      --------------------------------------CAGCAGCCGCGGTAATAC------------------------
2 Klenkia_terrae-IHUMI26.3_Scaffold4 100.0%   3.5%      GACGGTACCTGCAGAAGAAGCACCGGCCAACTACGTGCCAGCAGCCGCGGTAATACGTAGGGTGCAAGCGTTGTCCGGAA

                                        cov    pid   81          .         1         .         .         .         .         :         . 160
1 536F-Rp2                           100.0% 100.0%      --------------------------------------------------------------------------------
2 Klenkia_terrae-IHUMI26.3_Scaffold4 100.0%   3.5%      TTATTGGGCGTAAAGAGCTCGTAGGCGGTTTGTCGCGTCGGCTGTGAAAGCCCGGAGCTCAACTCCGGGTCTGCAGTCGA

                                        cov    pid  161          .         .         .         2         .         .         .         . 240
1 536F-Rp2                           100.0% 100.0%      --------------------------------------------------------------------------------
2 Klenkia_terrae-IHUMI26.3_Scaffold4 100.0%   3.5%      TACGGGCAGACTTGAGTGTTGCAGGGGAGACTGGAATTCCTGGTGTAGCGGTGAAATGCGCAGATATCAGGAGGAACACC

                                        cov    pid  241          :         .         .         .         .         3         .         . 320
1 536F-Rp2                           100.0% 100.0%      --------------------------------------------------------------------------------
2 Klenkia_terrae-IHUMI26.3_Scaffold4 100.0%   3.5%      GGTGGCGAAGGCGGGTCTCTGGGCAACAACTGACGCTGAGGAGCGAAAGCGTGGGGAGCGAACAGGATTAGATACCCTGG

                                        cov    pid  321          .         .         :         .         .         .         .         4 400
1 536F-Rp2                           100.0% 100.0%      --------------------------------------------------------------------------------
2 Klenkia_terrae-IHUMI26.3_Scaffold4 100.0%   3.5%      TAGTCCACGCCGTAAACGTTGGGCGCTAGGTGTGGGGGCCATTCCACGGTCTCCGTGCCGCAGCTAACGCATTAAGCGCC

                                        cov    pid  401          .         .         .         .         :         .         .         . 480
1 536F-Rp2                           100.0% 100.0%      --------------------------------------------------------------------------------
2 Klenkia_terrae-IHUMI26.3_Scaffold4 100.0%   3.5%      CCGCCTGGGGAGTACTGCCGCTACGCCAATACTCAAAGGAATTGACGGGGGCTCGCACAAGCGGCGGAGCATGTTGCTTA

                                        cov    pid  481          .         5         .         .         .         .         :         . 560
1 536F-Rp2                           100.0% 100.0%      --------------------------------------------------------------------------------
2 Klenkia_terrae-IHUMI26.3_Scaffold4 100.0%   3.5%      ATTCGATGCAACGCGAAGAACCTTACCTAGGCTTGACATGTGCGGAAATCCTCCAGAGATGGTGGGTCCGTAAGGGTCGC

                                        cov    pid  561          .         .         .         6         .         .         .         . 640
1 536F-Rp2                           100.0% 100.0%      --------------------------------------------------------------------------------
2 Klenkia_terrae-IHUMI26.3_Scaffold4 100.0%   3.5%      ACACAGGTGGTGCATGGTTGTCGTCAGCTCGTGTCGTGAGATGTTGGGTTAAGTCCCGCAACGAGCGCAACCCTCGTTCC

                                        cov    pid  641          :         .         .         .         .         7         .         . 720
1 536F-Rp2                           100.0% 100.0%      --------------------------------------------------------------------------------
2 Klenkia_terrae-IHUMI26.3_Scaffold4 100.0%   3.5%      ATGTTGCCAGCACGTCATGGTGGGGACTCATGGGAGACTGCCGGGGTCAACTCGGAGGAAGGTGGGGATGACGTCAAATC

                                        cov    pid  721          .         .         :         .         .         .         .         8 800
1 536F-Rp2                           100.0% 100.0%      --------------------------------------------------------------------------------
2 Klenkia_terrae-IHUMI26.3_Scaffold4 100.0%   3.5%      ATCATGCCCCTTATGTCTAGGGCTGCAAACATGCTACAATGGCCGGTACAAAGGGCTGCGATACCGCGAGGTGGAGCGAA

                                        cov    pid  801          .         .         .         .         :         .         .         . 880
1 536F-Rp2                           100.0% 100.0%      --------------------------------------------------------------------------------
2 Klenkia_terrae-IHUMI26.3_Scaffold4 100.0%   3.5%      TCCCAAAAAGCCGGTCTCAGTTCGGATTGGGGTCTGCAACTCGACCCCATGAAGTTGGAGTCGCTAGTAATCGCAGATCA

                                        cov    pid  881          .         9         .         .         .         .         :         . 960
1 536F-Rp2                           100.0% 100.0%      --------------------------------------------------------------------------------
2 Klenkia_terrae-IHUMI26.3_Scaffold4 100.0%   3.5%      GCAACGCTGCGGTGAATACGTTCCCGGGCCTTGTACACACCGCCCGTCACGTCACGAAAGTCGGTAACGCCCGAAGCCGG

                                        cov    pid  961          .         .         .         0         .         .         .         . 1040
1 536F-Rp2                           100.0% 100.0%      -------------------------------------------------------AAGTCGTAACAAGGTAGCCG-----
2 Klenkia_terrae-IHUMI26.3_Scaffold4 100.0%   3.5%      TGGCCCAACCCCTTGTGGGAGGGAGCCGTCGAAGGCGGGATCGGCGATTGGGACGAAGTCGTAACAAGGTAGCCGTACCG

                                        cov    pid 1041          :         .         .         .   ] 1084
1 536F-Rp2                           100.0% 100.0%      --------------------------------------------
2 Klenkia_terrae-IHUMI26.3_Scaffold4 100.0%   3.5%      GAAGGTGCGGCTGGATCACCTCCTTTCTAAGGAGCACTGGCCGG
```
